# First Quarterly Meeting West Texas Medical Association; Minutes

**Published:** 1889-09

**Authors:** D. Berrey

**Affiliations:** Secretary


### SOCIETY NOTES.

### MINUTES OF THE FIRST QUARTERLY MEETING OF THE WEST TEXAS MEDICAL ASSOCIATION.

The first quarterly meeting of the West Texas Medical Association was held at Scholz's hall July 31, at 10 a. m. The President, Dr. P. W. Johns being absent, Vice President Cross was called to the chair. Dr. D. Berrey secretary. Present Drs. Cupples, Chew, Cross, Crisp, of Batesville, Texas; Tutwaler, of Flatonia; Spring, Trollinger, Howard, of Cotulla; Hertzberg, Fennell, of Seguin; Kingsley, King, Oldham, Field, Hardee, of Pleasanton; McGirk, Williams, of Devine; Watts and Berrey. Dr. Cross stated the object of the meeting, which was to organize quarterly meetings of the West Texas Medical Association, and asked the hearty co-operation of the profession of the city and surrounding country in our efforts.

The minutes of the last monthly meeting of the Association were read and adopted.

Dr. Kingsley moved that on account of the central location of San Antonio, the quarterly sessions of the society be held regularly in this city.

Dr. Cupples amended Kingsley's motion to read that the first two quarterly sessions be held in San Antonio, and the location of the other two places of meeting be left optional with the association. Amendment accepted and carried.

Dr. Berrey moved that the next quarterly meeting of the association be held conjointly with the regular annual meeting of the West Texas Medical Association on the last Wednesday in October. Carried. (This motion was made with the view of having members from the country present at the annual election of officers).

The following gentlemen were elected to membership: N. E. Field, M. D., San Antonio; N. H. McGirk, M. D., San Antonio; S. Burg, M. D., San Antonio; and Richard Dart, M. D., Brackettville.

Drs. Howard, of Devine, Williams, of Cotulla, Tutwaler, of Flatonio, and Crisp, of Zavalla county, filed applications for membership with the board of censors. After diicussing some routine business the association adjourned till 5 p. m.

Evening session; meeting called to order at 5 p. m., at Scholz's hall. The President Dr. P. W. Johns in the chair. Dr. D. Berrey secretary. The president called for written articles, and Dr. J. M. Hays presented a paper on antiseptic surgery, which was fully discussed. Dr. Berrey moved that Dr. Hays be tendered the thanks of the Association for his paper, and that it be forwarded to Dăniel's Texas Medical Journal for publication. Dr. Spring presented a most interesting paper on abscess of the liver. Dr. Cupples opened the discussion and gave the Association a resume of his vast experience. Almost every one present took part in the discussion, and many valuable hints in the diagnosis, prognosis, and treatment of abscess of the liver were elicited. Dr. Spring received the hearty thanks of the Association for his valuable paper, and it was ordered published.

D. Berrey, M. D., Secretary.

